# Recovery from an Acute Infection in *C. elegans* Requires the GATA Transcription Factor ELT-2

**DOI:** 10.1371/journal.pgen.1004609

**Published:** 2014-10-23

**Authors:** Brian Head, Alejandro Aballay

**Affiliations:** Department of Molecular Genetics and Microbiology, Duke University Medical Center, Durham, North Carolina, United States of America; Genentech, United States of America

## Abstract

The mechanisms involved in the recognition of microbial pathogens and activation of the immune system have been extensively studied. However, the mechanisms involved in the recovery phase of an infection are incompletely characterized at both the cellular and physiological levels. Here, we establish a *Caenorhabditis elegans*-*Salmonella enterica* model of acute infection and antibiotic treatment for studying biological changes during the resolution phase of an infection. Using whole genome expression profiles of acutely infected animals, we found that genes that are markers of innate immunity are down-regulated upon recovery, while genes involved in xenobiotic detoxification, redox regulation, and cellular homeostasis are up-regulated. *In silico* analyses demonstrated that genes altered during recovery from infection were transcriptionally regulated by conserved transcription factors, including GATA/ELT-2, FOXO/DAF-16, and Nrf/SKN-1. Finally, we found that recovery from an acute bacterial infection is dependent on ELT-2 activity.

## Author Summary

Infections by bacterial pathogens often produce substantial tissue damage and alter metabolism in the host that, if left unchecked, could be detrimental to overall fitness. The cellular and systemic responses that resolve these alterations in the host are not well defined. Here, we examine transcriptional networks in an animal host that are modulated during the resolution phase of an intestinal infection treated with an antibiotic. Up-regulation of genes involved in detoxification and cellular homeostasis during the resolution phase is controlled by the conserved endodermal GATA transcription factor ELT-2. GATA transcription factors are known to be involved in the development, differentiation, and function of a diverse array of metazoan tissue types. Therefore, our results ascribe a new role to GATA transcription factors in recovery from an acute infection. Fully characterizing the host response during resolution of an infection will lead to a better understanding of human health concerns related to recurrent infections, wound healing, autoimmune diseases, and chronic inflammatory disorders.

## Introduction

The course of human bacterial infections is controlled by a combination of immune responses, physiological changes, and, if necessary, antibiotic treatment. To recover from an infection and return to homeostasis, the host must activate mechanisms capable of controlling the damage caused by pathogen virulence factors, inflammation, and a potentially toxic antibiotic exposure. If these alterations in host physiology are not handled appropriately, the host risks entering a state of reduced fitness. This reduced fitness manifests in the form of recurrent infections, inappropriate wound healing, autoimmune diseases, and chronic inflammatory disorders. While the mechanisms involved in the recognition of microbial pathogens as such and the subsequent activation of the immune system have been extensively studied, the pathways involved in host recovery after an infection remain understudied.

To examine the biological changes that take place during the recovery phase of an acute bacterial infection, we decided to use the nematode *Caenorhabditis elegans* as a simple model host. Various human bacterial pathogens, including *Pseudomonas aeruginosa*, *Salmonella enterica*, *Staphylococcus aureus*, and *Enterococcus faecalis*, have been shown to colonize and kill *C. elegans* using conserved virulence mechanisms [1]–[4]. Moreover, *C. elegans* responds to infections using an inducible innate immune system that is controlled by several evolutionary conserved signaling cascades including the p38-MAPK (PMK-1), insulin-IGF (DAF-16), GATA (ELT-2), and TGF-B (SMA-6) pathways [5]–[8]. It is therefore likely that investigating *C. elegans* recovery from bacterial infection would shed light on host responses that reestablish homeostasis post-infection.

In this study, we established a *C. elegans-S. enterica* pathogenesis system as a model of acute infection by infecting nematodes with *S. enterica* and subsequently resolving the infection by treatment with the antibiotic Tetracycline. Using this acute infection model, we profiled gene expression changes in the host over the course of the infection and during the recovery phase of the infection. We found that during recovery, certain components of the host innate immune response were dampened, while mechanisms involved in xenobiotic detoxification, redox regulation, and cytoprotection were activated. A large number of the genes altered during recovery corresponded to intestinal genes regulated by ELT-2, which is a conserved GATA transcription factor that plays a key role in the control of intestinal functions in *C. elegans*. Further studies indicated that the recovery from acute *S. enterica* infection required ELT-2, indicating that ELT-2 controls not only induction of innate immune response genes but also genes that play a crucial role in the resolution of an infection.

## Results

### Use of a *C. elegans-S. enterica* pathogenesis system to model acute infections

Although host responses that limit microbial infection have been extensively studied, the mechanisms involved in the recovery phase of an infection are incompletely characterized at both the cellular and physiological level. We decided to use *Caenorhabditis elegans* as a simple model host for assessing biological changes during the recovery phase from an acute infection. *C. elegans* is propagated in the laboratory by feeding them *E. coli* strain OP50. *E. coli* is effectively disrupted by the *C. elegans* pharyngeal grinder and essentially no intact bacterial cells can be found in the intestinal lumen of young, immunocompetent animals. However, pathogenic bacteria such as *Salmonella enterica* are capable of killing *C. elegans* by infectious processes that correlate with the accumulation of bacteria in the intestine. As in mammalian hosts, a small inoculum of *S. enterica* is capable of establishing a persistent infection in *C. elegans* that does not require constant exposure to bacteria and cannot be prevented by transferring the infected animals to plates containing *E. coli* [2], [9].

To determine whether a long-lasting, chronic *S. enterica* infection could be easily reversed by antibiotic treatment to model a short, acute infection we used *fer-1(b232ts)* animals, which are fertilization defective at the restrictive temperature. This prevents losing track of the initially infected animals in the morass of progeny that would be otherwise generated following an acute infection. We first established that transferring *S. enterica-*infected animals to plates containing the bacteriostatic antibiotic Tetracycline and seeded with Tet^R^ *E. coli* was sufficient to significantly reduce bacterial burden (Figure S1). Subsequently, we decided to use 50 µg/ml Tetracycline treatment to reduce *S. enterica* burden to model an acute infection in *C. elegans*.

**Figure 1:**
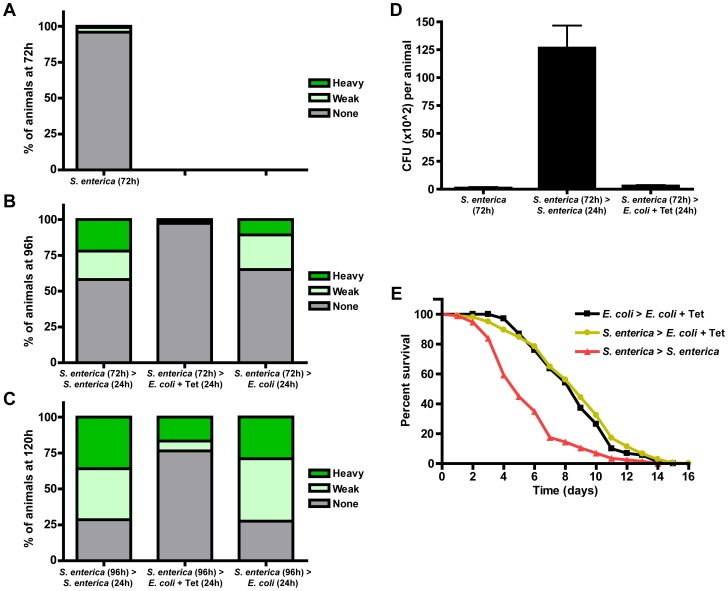
Tetracycline treatment models *S. enterica* acute infection in *C. elegans*. (A) *fer-1(b232ts)* L1 animals were exposed to *S. enterica*—GFP for 72 hours. (B) *fer-1(b232ts)* L1 animals were exposed to *S. enterica*—GFP for 72 hours and transferred to the indicated plates for 24 hours. (C) *fer-1(b232ts)* L1 animals were exposed to *S. enterica*—GFP for 96 hours and transferred to the indicated plates for 24 hours. At each time point, overall GFP intensity in the intestinal lumen was determined using an MZFLIII Leica stereomicroscope. Three levels of colonization were determined as heavy, weak, or none as described in Materials and Methods. N = 104–150 animals per condition. The graph represents the combined results of 2 independent experiments. (D) Quantification of colony forming units of *fer-1(b232ts)* L1 animals exposed to *S. enterica*—GFP for 72 hours, *S. enterica*—GFP for 96 hours, or *S. enterica*—GFP for 72 hours and then treated with Tetracycline for 24 hours. N = 10 animals per condition. The graph represents the combined results of 4 independent experiments. (E) *fer-1(b232ts)* L1 animals were exposed to *E. coli* or *S. enterica*—GFP for 72 hours and then transferred to *E. coli* plus Tetracycline or *S. enterica—*GFP. Animals were scored for survival 72 hour post initial exposure to *S. enterica*. N = 60 animals per condition. The graph represents the combined results of 5 independent experiments.

We monitored bacterial accumulation over the course of a 120 hour infection in synchronized larval stage 1 (L1) *fer-1(b232ts)* animals continuously grown on plates seeded with *S. enterica-*GFP or transferred to Tetracycline-containing plates seeded with Tet^R^ *E. coli*. Consistent with previous findings indicating that *C. elegans* larvae are highly resistant to pathogen-mediated killing and that death does not occur during the first several days of an *S. enterica* infection [9], [10], we observed that only 4.1% of the animals exposed to *S. enterica-*GFP starting at the L1 stage were colonized 72 hours later (Figure 1A). In contrast, at 96 and 120 hours post-exposure, 41.9% and 71.4% of the animals were colonized by *S. enterica*-GFP (Figure 1B–C). We found that transferring animals from *S. enterica* at 72 or 96 hours to Tetracycline-containing plates for 24 hours reduced bacterial burden (Figure 1B–C). Quantification of the number of live bacteria in animals that were infected with *S. enterica-*GFP for 72 hours and treated with Tetracycline for 24 hours showed a significant reduction of bacterial burden (Figure 1D), confirming that Tetracycline treatment can prevent *S. enterica* from persistently colonizing the *C. elegans* intestine and causing a chronic infection.

Even though our results indicate that Tetracycline can prevent *S. enterica* from causing a persistent colonization of the *C. elegans* intestine, it was unclear whether acute pathogenic challenge would damage the animal and translate into an associated reduction in survival. As shown in Figure 1E, we found that the survival of animals infected with *S. enterica* and then treated with Tetracycline is significantly higher than that of animals continuously infected (Figure 1E; yellow vs. red lines). Also, survival of infected and then Tetracycline-treated animals is nearly equivalent to animals that were never infected (Figure 1E, yellow vs. black lines). Treatment with Tetracycline in the presence of killed bacteria only increased *C. elegans* mean lifespan from 14.2 to 14.9 days (Figure S2). Taken together, these studies show that an *S. enterica* infection can be resolved by treating the animals with Tetracycline and indicate that this type of treatment can be used to model an acute *S. enterica* infection that progresses towards chronicity if the animals were to remain untreated.

### Recovery from an acute *S. enterica* infection results in the down-regulation of immune responses and up-regulation of cellular homeostatic mechanisms

**Figure 2:**
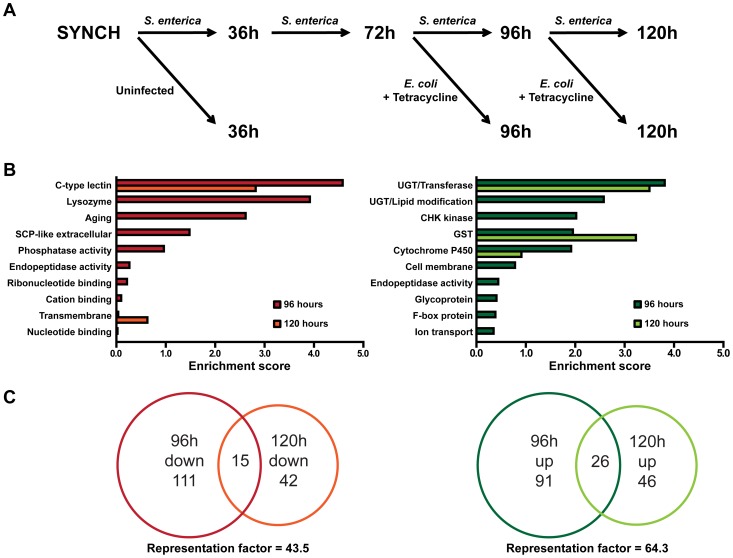
Whole genome expression analysis reveals down-regulated immune response genes and up-regulated detoxification genes during resolution of acute *S. enterica* infection. (A) Flowchart of animal cohorts collected for the microarray analysis. (B) Gene ontology analysis of genes regulated during recovery at the 96 and 120 hour time points using the DAVID Bioinformatics Database. Enrichment scores of the 96-hour and 120-hour down-regulated clusters are shown in the left panel. Enrichment scores of the 96-hour and 120-hour up-regulated clusters are shown in the right panel. (C) Venn diagrams showing the overlap of the 96-hour and 120-hour down-regulated genes, left, and overlap of the 96-hour and 120-hour up-regulated genes, right. Representation factors are 43.5 and 64.3, respectively.

To investigate cellular mechanisms potentially involved in recovery after an infection, we utilized Agilent *C. elegans* gene expression microarrays to identify changes in gene expression during infection and changes that take place after the infection is reversed by treatment with Tetracycline (Figure 2A, Tables S1 and S2). Initially, we focused our analysis on animals that were infected with *S. enterica* for 96 hours vs. animals that were infected for 72 hours and treated with Tetracycline for 24 hours to resolve the infection. At 96 hours, 99% of the animals were alive in both conditions (Figure 1E, Day 1). Overall, 243 genes, or approximately 1% of the *C. elegans* genome, were altered more than 2-fold (p<0.05) when comparing the 96-hour cohorts. Of these altered genes, 126 were down-regulated and 117 were up-regulated (Table S2).

To identify related gene groups that are transcriptionally controlled by pathways potentially involved in the changes that take place after infection, we performed an unbiased gene enrichment analysis using the database for annotation, visualization and integrated discovery (DAVID, http://david.abcc.ncifcrf.gov/) [11]. The 10 gene ontology (GO) clusters with the highest DAVID enrichment score are shown in Figure 2B and Table S3. For the subset of down-regulated genes that respond to the resolution of the infection, the 2 top-scoring GO clusters, c-type lectins and lysozyme groupings, have previously been described as part of an inducible *C. elegans* immune response to a variety of pathogens [12]–[15]. For the subset of up-regulated genes that respond to the resolution of the infection, 4 of the top 10 highest scoring ontology clusters are associated with xenobiotic detoxification, redox regulation, or cytoprotection [16], [17]. These results indicate that the activation of the innate immune system of *C. elegans* by *S. enterica* infection is attenuated once the infection is resolved and that certain cellular homeostatic pathways are activated during recovery.

Since only 42% of the animals exposed to *S. enterica* for 96 hours exhibited visible bacterial colonization (Figure 1B), we decided to examine gene expression profiles of animals that were infected for 120 hours, which exhibited an even higher degree of bacterial colonization (Figure 1C). A comparison of gene expression profiles from animals that were infected with *S. enterica* for 120 hours vs. animals that were infected for 96 hours and treated with Tetracycline for 24 hours identified 57 and 72 genes that are down- or up-regulated greater than 2-fold (p<0.05), respectively (Table S2). Analysis of GO terms in these gene sets via DAVID gives a shorter but similar list of enriched gene clusters (Figure 2B and Table S3), confirming that, as the infection resolves, marker genes of immune activation are down-regulated while genes that correspond to cellular homeostatic pathways are up-regulated. Moreover, the significant overlap between 96 and 120 hour treatment gene sets indicates that the changes that take place after an infection is resolved are reproducible and that similar transcriptional profiles are elicited at different times (Figure 2C and Table S4). The smaller number of genes down- and up-regulated by the resolution of the infection at 120 hours compared to 96 hours could be a consequence of the higher heterogeneity of *S. enterica* colonization in the 120-hour population (comparison of Figures 1B and 1C). It is also possible that as the infection progresses, the animals suffer irreversible damage that makes them less responsive to antibiotic treatment.

**Figure 3:**
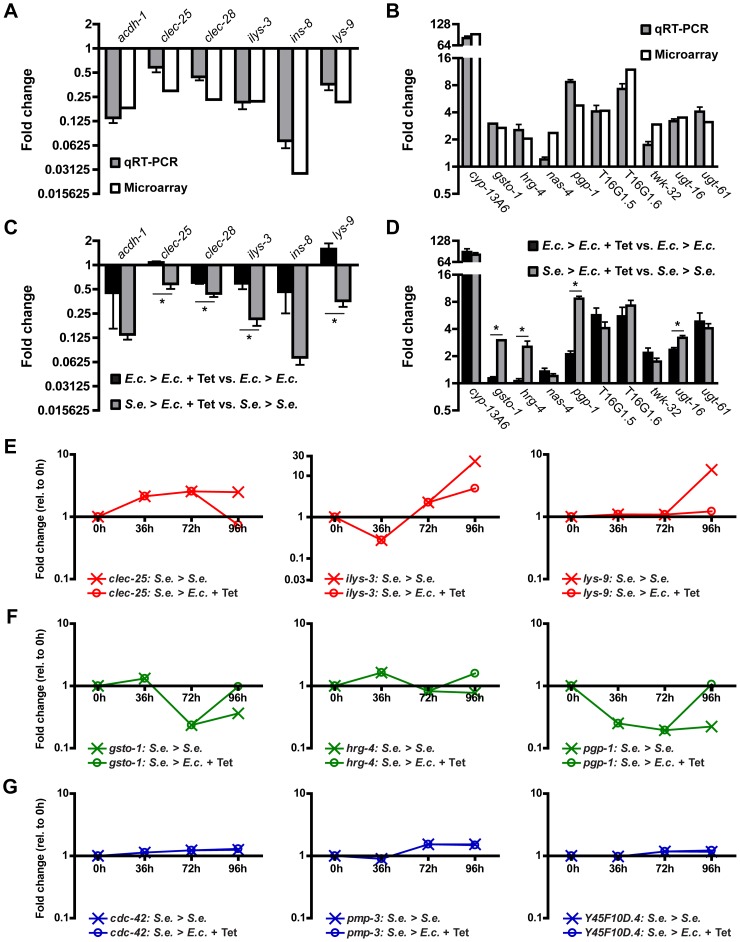
Gene expression changes in infected animals treated with Tetracycline. (A–B) Transcript levels of 6 selected down-regulated genes (A) and 10 selected up-regulated genes (B) from L1 animals grown on *S. enterica* for 72 hours and then treated with Tetracycline for 24 hours relative to L1 animals grown on *S. enterica* for 96 hours. Out of the 17 studied genes, only the 16 validated genes are shown. Gray bars represent fold change as determined using qRT-PCR. White bars represent fold change as determined using microarrays. (C–D) Transcript levels of 6 selected down-regulated genes (C) and 10 selected up-regulated genes (D) as determined using qRT-PCR. Black bars represent gene expression changes in L1 animals grown on *E. coli* for 72 hours and then treated with Tetracycline for 24 hours relative to L1 animals grown on *E. coli* for 96 hours. Gray bars represent gene expression changes in L1 animals grown on *S. enterica* for 72 hours and then treated with Tetracycline for 24 hours relative to animals grown on *S. enterica* for 96 hours. qRT-PCR studies were performed in triplicate. SEM is shown. Statistical significance is indicated (p<0.05: *). (E–G) Transcript levels of 3 selected down-regulated genes (E), 3 selected up-regulated genes (F), and 3 selected housekeeping genes (F) over the infection time course. The expression values of animals grown on *S. enterica* for 72 hours and then treated with Tetracycline for 24 hours are denoted with open circles. The expression values of animals grown on *S. enterica* for 96 hours are denoted with an X.

To validate the results of the microarrays, we performed quantitative real-time PCR (qRT-PCR) on a subset of the 243 genes that change upon Tetracycline treatment of infected animals. This subset includes 11 up-regulated genes and 6 down-regulated genes that were either present in a high scoring GO cluster, were highly misregulated, or both. We performed qRT-PCR on RNA harvested from *C. elegans* that were subjected to the same conditions as in the microarray studies. As shown in Figure 3A and 3B, the changes in gene expression as assessed by qRT-PCR were comparable to those observed by microarray profiling. Further analysis indicated that 16 of the 17 genes had statistically significant expression changes during Tetracycline-mediated recovery from *S. enterica* infection (Figure 3A and 3B). Thus, the microarray data accurately reflects the majority of gene expression differences between treated and non-treated animals.

Resolution of *S. enterica* infection by treatment with Tetracycline results in the down-regulation of genes that are markers of innate immunity and the up-regulation of genes that function in xenobiotic detoxification, redox regulation, and cytoprotection (Figure 2B). While the resolution of the infection may be responsible for altering the expression of these genes, it is also possible that Tetracycline is directly inducing these changes. To distinguish between these two possibilities, we compared changes in gene expression due to Tetracycline alone vs. changes in gene expression due to recovery from infection by treatment with Tetracycline. We found that expression of 8 out of 16 tested genes were significantly different (Figure 3C–D), highlighting the role of these 8 recovery genes in pathways that are altered during the resolution of the *S. enterica* infection. Considering that the genome wide microarray shows that 243 genes change their expression upon recovery at 96 hours, we estimate that approximately 122 genes are regulated by recovery from infection independently of Tetracycline while the remaining genes are regulated by the inclusion of Tetracycline alone. This suggests that Tetracycline may be directly inducing gene expression changes in the host that may help clear an infection independently of its antimicrobial activity.

To confirm the finding that a subset of genes is altered upon resolution of an *S. enterica* infection independent of Tetracycline, we performed equivalent experiments using the antibiotic Kanamycin. These studies indicate that Kanamycin alone did not alter the expression of 9 tested genes in uninfected animals (Figure S3A–B). Furthermore, 8 of the 9 alterations in gene expression seen in infected animals treated with Kanamycin are similar to those seen in infected animals treated with Tetracycline (Figure S3C–D).

To provide further insight into the behavior of genes altered during antibiotic-mediated recovery, we examined gene expression profiles over the course of the 96-hour infection (Table S1). We focused our analysis on qRT-PCR-confirmed genes that are known markers of immune activation and genes that correspond to cellular homeostatic pathways. The expression of innate immunity genes diminished significantly after the infection was resolved by Tetracycline treatment (Figure 3C and E). In contrast, genes involved in regulating cellular homeostasis were significantly up-regulated upon recovery from infection (Figure 3D and F). As a control, the expression of 3 select housekeeping genes remained relatively constant both during the course of the infection and during recovery (Figure 3G). Overall, these studies suggest that as the infection resolves, cellular homeostatic mechanisms are activated while elements of the immune response are attenuated.

### The GATA transcription factor ELT-2 is required for the resolution of the *S. enterica* infection

Several of the GO clusters identified in the set of genes up-regulated during resolution of the infection correspond to genes whose products are involved in detoxification. We therefore hypothesized that the reduction of the pathogenic insult during the recovery phase of an infection may trigger processes involved in detoxification and clearance of immune effectors that, while necessary to combat pathogens, can have deleterious effects on the host. Recently, it was demonstrated that reactive oxygen species (ROS), a component of the *C. elegans* immune response to *S. enterica* and other pathogens [18]–[20], contributes to infectious pathogenicity (i.e., damage to the host). Thus, we decided to study *gsto-1*, which is an up-regulated gene that encodes an omega-class glutathione S-transferase that protects *C. elegans* from oxidative stress under non-infected conditions [21]. We found that survival of *gsto-1(RNAi)* animals infected with *S. enterica* and treated with Tetracycline was not significantly different from that of control animals (Figure S4).

**Figure 4:**
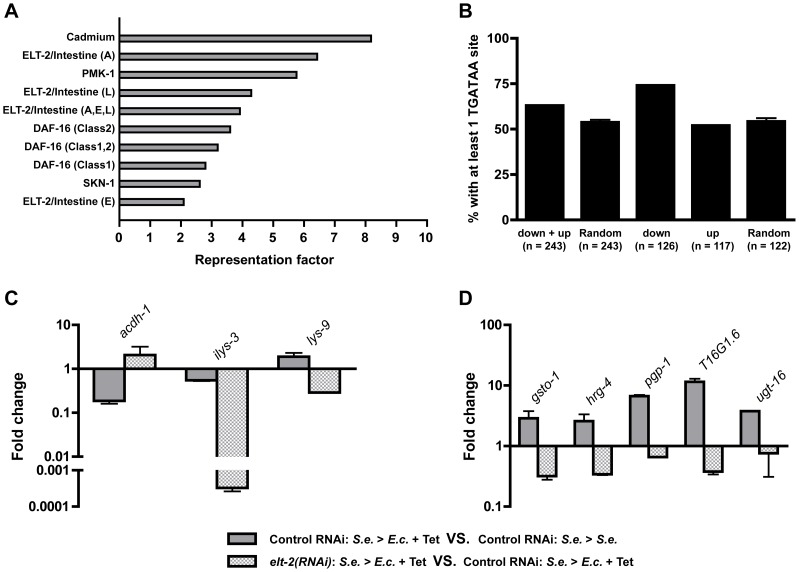
ELT-2 regulates the expression of specific genes during recovery. (A) Representation factors of previously studied data sets that overlap with genes altered during recovery at the 96-hour time point. (B) Percentage of down- and up-regulated recovery genes with at least one TGATAA site in the 1.5 kb sequence upstream of the transcriptional start site. Randomly selected gene sets are included as controls. (C–D) Transcript levels of 3 selected down-regulated genes (C) and 5 selected up-regulated genes (D) as determined using qRT-PCR. Gray bars represent gene expression changes in control *fer-1(b232ts)* young adult animals exposed to *S. enterica*—GFP for 36 hours and then treated with Tetracycline for 24 hours relative to control *fer-1(b232ts)* young adult animals grown on *S. enterica*—GFP for 60 hours. Checkered gray bars represent gene expression changes in *fer-1(b232ts) elt-2(RNAi)* young adult animals exposed to *S. enterica* for 36 hours and then treated with Tetracycline for 24 hours relative to control *fer-1(b232ts)* young adult animals exposed to *S. enterica*—GFP for 36 hours and then treated with Tetracycline for 24 hours. qRT-PCR studies were performed in duplicate. SEM is shown.

The lack of a significant effect by *gsto-1* RNAi could be attributed to incomplete RNAi or to functional redundancy among the multitude of detoxification genes that are up-regulated during recovery (Figure 2B and Table S3). The *gsto-1* locus is transcriptionally regulated by the GATA transcription factor ELT-2 [21], leading us to consider a role for ELT-2 in controlling the expression of a set of genes required for resolution of an infection. Consequently, we applied several *in silico* approaches to determine whether ELT-2-regulated genes are present in the genes whose expression changes by the resolution of an infection. We compared the set of genes altered during recovery to previously identified ELT-2-regulated gene sets and to other control data sets. The ELT-2-regulated gene sets were among the 10 data sets with the strongest overlap with our recovery gene set (Figure 4A and Table S5). As ELT-2 regulates the expression of genes in the *C. elegans* intestine via trans-acting activity at TGATAA (extended GATA) cis-regulatory motifs [22], [23], we looked for the presence of TGATAA binding sites in the putative promoter regions of the down- and up- regulated genes. Approximately 63% of the 243 genes regulated by recovery contain at least 1 TGATAA site within the 1.5 kb sequence upstream of their transcriptional start site (Figure 4B). By comparison, only 54% of genes in 3 randomly selected gene sets (n = 243 each) have at least 1 TGATAA sequence in the equivalent 1.5 kb region (Figure 4B). Additionally, we observed that at least 1 TGATAA site is present in the putative promoter region of 7 of the 8 recovery genes verified by qRT-PCR (Table S6). While *gsto-1* does not contain a TGATAA site in this 1.5 kb region, it does have a single site 3.8 kb upstream of the transcriptional start site. Moreover, it has been experimentally demonstrated that ELT-2 regulates the transcription of *gsto-1* [21].

Consistent with the post-developmental role of ELT-2 in the regulation of adult intestinal functions [5], [6], [24]–[26], another *in silico* approach showed that 17 out of 32 (53%) recovery genes with at least 1 TGATAA site and for which the data is available are expressed in the intestine (Table S7). Only 1 out of these 32 genes is expressed in the hypodermis where other GATA transcription factors function [27]. This analysis also showed that 4 out of 8 recovery genes verified by qRT-PCR are expressed in the intestine (Table S7). Taken together, our *in silico* analyses leads to the hypothesis that ELT-2 controls the expression of a subset of genes during the recovery phase of an infection.

To further substantiate a role for ELT-2 in the transcriptional regulation of genes during recovery, we studied the effect of *elt-2* RNAi on the expression of recovery genes. As ELT-2 is essential for *C. elegans* larval development [22], RNAi was performed on late larval stage 4 (L4) animals. This approach has been used successfully to inhibit *elt-2* expression for at least 6 days [28], [29]. As shown in Figure 4D, RNAi of *elt-2* inhibited the expression of the 5 studied genes that are up-regulated during recovery from the infection by treatment with either Tetracycline (Figure 3D) or Kanamycin (Figure S3B). Inhibition of *elt-2* by RNAi also further down-regulated the expression of *ilys-3* and *lys-9* (Figure 4C). However, RNAi of *elt-2* does not result in the unselective down-regulation of recovery genes as *acdh-1* is not down-regulated (Figure 4C). In addition, certain ELT-2-controlled immunity and structural genes [12], [28] are not significantly altered during recovery from *S. enterica* infection (Figure S5A–B). We further confirmed by qRT-PCR that transcript levels of *clec-67*, which is a known marker of immunity controlled by ELT-2 [12], are not altered upon recovery (Figure S5C). We conclude that expression of a specific intestinal gene program during resolution of an infection is dependent upon the action of the GATA transcription factor ELT-2.

**Figure 5:**
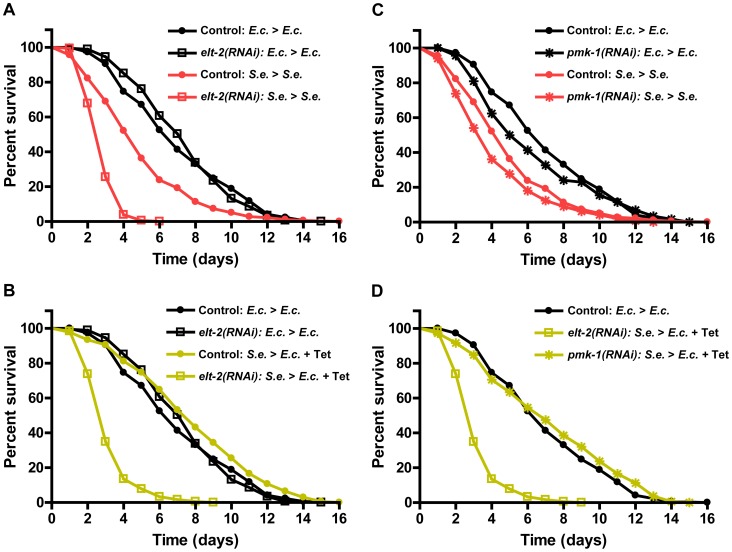
*elt-2(RNAi)* animals are unable to resolve an infection. (A) Control *fer-1(b232ts)* or *fer-1(b232ts) elt-2(RNAi)* young adult animals were exposed to *E. coli* or *S. enterica*—GFP for 36 hours and then transferred to *E. coli* or *S. enterica*—GFP and scored for survival. (B) Control *fer-1(b232ts)* or *fer-1(b232ts) elt-2(RNAi)* animals were exposed to *E. coli* or *S. enterica*—GFP for 36 hours and then transferred to *E. coli* or *E. coli* plus Tetracycline and scored for survival. (C) Control *fer-1(b232ts)* or *fer-1(b232ts) pmk-1(RNAi)* young adult animals were exposed to *E. coli* or *S. enterica*—GFP for 36 hours and then transferred to *E. coli* or *S. enterica*—GFP and scored for survival. (D) Control *fer-1(b232ts)* or *fer-1(b232ts) pmk-1(RNAi)* animals were exposed to *E. coli* or *S. enterica*—GFP for 36 hours and then transferred to *E. coli* or *E. coli* plus Tetracycline and scored for survival. N = 60 animals per condition. The graphs represent the combined results of 3 independent experiments.

To test whether ELT-2 is required for recovery after infection, we studied the survival of *elt-2(RNAi)* animals infected with *S. enterica* and treated with Tetracycline. RNAi inhibition of *elt-2* starting at the L4 stage did not alter the survival of animals growing on live *E. coli* (Figure 5A; black lines), nor did it alter survival in the presence of Tetracycline (Figure S6). This data indicates that L4 *elt-2(RNAi)* animals are not sick merely due to disruptions in basal immunity or intestinal function. However, RNAi of *elt-2* prevented the recovery of infected animals by treatment with Tetracycline (Figure 5B; yellow lines), highlighting the role of ELT-2 during the recovery phase of the infection. In agreement with previously published reports that ELT-2 regulates innate immunity [12], [28], RNAi of *elt-2* did significantly reduce survival of animals continuously infected with *S. enterica* (Figure 5A; red lines). To address whether genes crucial for immunity are generally required for recovery, we studied *pmk-1*, which encodes a p38 mitogen-activated protein kinase that is a major regulator of innate immunity in *C. elegans* [14], [30], [31]. Even though RNAi of *pmk-1* elicited sensitivity to *S. enterica-*mediated killing (Figure 5C; red lines), it did not prevent the recovery of infected animals by treatment with Tetracycline (Figure 5D). Taken together, these results indicate that ELT-2 is required for both early immune responses against pathogens and responses that are activated upon recovery from an infection by *S. enterica*.

## Discussion

Using gene expression profiling, *in silico* analysis, and reverse genetic approaches, we have defined a novel post-developmental role for the GATA transcription factor ELT-2 during the resolution of an infection. ELT-2 was originally identified as a key regulator of *C. elegans* intestinal specification during development [22]. However, it is now becoming clear that ELT-2 has an extensive post-developmental role in the regulation of a plethora of adult intestinal functions. Under the control of ELT-2, the 20 cells of the adult intestine in *C. elegans* function in nutrient uptake, synthesis and storage of macromolecules, epithelial immunity, and host-microbial communication [5], [6], [24]–[26]. There are several additional GATA transcription factors encoded in the *C. elegans* genome, including ELT-4 and ELT-7, which regulate intestinal gene expression programs [32]. Further studies will be required to determine their possible contribution to the recovery process.

Owing to the multi-functional nature of the intestine and due to the fact that ELT-2 regulates nearly all intestinal genes [33], it is not surprising that half of the genes in the *C. elegans* genome have putative ELT-2 binding sites (Figure 4B). Thus, it is logical to conclude that the specification of different functional outputs that takes place in the *C. elegans* intestine during the complete course of an infection is controlled by additional co-factors that act together with ELT-2. Recent work has demonstrated that GATA transcription factors, including ELT-2, act coordinately with the insulin-IGF pathway transcriptional regulator DAF-16 in a cell-autonomous manner to regulate lifespan extension in *C. elegans* [34]. It is therefore plausible that DAF-16 acts with ELT-2 to co-regulate genes important for infection resolution. Indeed, we observed a significant enrichment of both ELT-2- and DAF-16-controlled targets in our set of genes altered during recovery from infection (Figure 4A and Table S5). An emerging theme is that coordinated transcriptional activity of DAF-16 and ELT-2 would be necessary for the modulation of cytoprotective pathways that, in turn, are required for a majority of cellular stress response pathways [16].

Another candidate factor that may regulate damage response genes in conjunction with ELT-2 and/or DAF-16 is the Nrf1/SKN-1 transcription factor. Previous work has demonstrated that signaling cascades downstream of reactive oxygen species (ROS) induce a cytoprotective SKN-1 pathway [35], [36]. SKN-1 might regulate cytoprotective genes downstream of or in parallel to ELT-2 and/or DAF-16 to mediate resolution of an infection. Indeed, SKN-1-positively regulated targets are significantly enriched in the set of genes that are up-regulated during infection resolution (Table S5).

Mounting evidence indicates that ELT-2 activity is modulated under a variety of environmental conditions or physiological states. ELT-2-mediated immunity to a variety of pathogens is activated by currently unknown mechanisms. Interestingly, a paper by Lee and colleagues demonstrates that the intestinal pathogen *B. pseudomallei* can actively target and degrade ELT-2 to prevent host immune responses [29]. We failed to observe any alterations in ELT-2 protein localization or abundance caused by *S. enterica* infection or recovery. These pathogens, which kill *C. elegans* at a distinctly different rate, must utilize different mechanisms to overcome the host immune system.

Signals during the initial decline in infection may function to reprogram the transcriptional activity of ELT-2 from an innate immune program to a cytoprotective one. These unidentified signals may be bacterial- and/or host-derived. Specific bacterial-derived signals, such as those involved in biofilm formation or quorum sensing, may be the primary trigger for the ELT-2 switch [26]. These bacterial-derived signals might act directly on ELT-2 or they may transit through host-encoded genes. Alternatively, host-encoded regulators that normally function during development, such as the END-1/END-3 specification factors, might be re-activated during the resolution of infection to direct the transcription of detoxification genes by ELT-2. Interestingly, the END-1/END-3 system lies downstream of the oxidative stress (ROS) response protein SKN-1 in the development of the *C. elegans* intestine [24]. Alternatively, changes in ELT-2 activity may be controlled by local chromatin remodeling in a manner similar to the regulation of DAF-16 transcriptional activity [37].

In summary, our results identified a new, key role for ELT-2 during recovery from a bacterial infection. We revealed that during recovery from an infection, genes that are markers of innate immunity are down-regulated, while the expression of genes involved in xenobiotic detoxification, redox regulation, and cytoprotection is enhanced. Interestingly, a number of genes encoding antibacterial factors (ABFs) are up-regulated during the course of the *S. enterica* infection (Table S1). However, the expression of *abf* genes is not down-regulated once the infection is resolved. This could be due to a mechanism used by *C. elegans* to maintain high levels of *abf* genes throughout reproductive adulthood. It is also possible that ABFs have a high specificity for damaging prokaryotic cells, having little or no impact on host cells. Thus, there would be no immediate need to reverse their up-regulation once the infection is resolved, unlike the case of lysozyme-encoding genes, which could potentially damage host cells. The ELT-2 interaction with the aforementioned co-factors may dictate the specificity of the expression profile during the different phases of an infection. A number of microbial killing pathways and cellular homeostatic pathways are controlled by the nervous system in infected *C. elegans* [38]–[46]. An important question that remains to be evaluated is whether the nervous system also plays a role in the control of the mechanisms involved in recovery after infections have been cleared.

## Materials and Methods

### Nematode and bacterial strains

*C. elegans* strain HH142 *fer-1(b232ts)* was provided by the *Caenorhabditis* Genetics Center. *C. elegans* were maintained at 15°C on NGM—OP50 plates without antibiotics. The following bacterial strains were used for experiments: *Escherichia coli* strain OP50-1 [Sm^R^] [47], *E. coli*—dsRed strain OP50 [Amp^R^, Cb^R^] [47], *E. coli* strain HT115 [Tet^R^] [48], *E. coli* strain HT115 pL4440 [Amp^R^, Tet^R^] [48], *E. coli* strain DH5α pSMC21 [Kan^R^] [49], *Salmonella enterica enterica* serovar *Typhimurium* strain 1344 [Sm^R^] [50], *S. enterica*—GFP strain SM022 [Sm^R^, Kan^R^] [51]. Bacteria were grown overnight for 14 hours in 3 ml LB broth at 37°C.

### Visualization of bacterial accumulation in the nematode intestine

*fer-1(b232ts)* animals were synchronized by treating gravid adults with sodium hydroxide and bleach. About 2,000 synchronized L1 animals were grown on full lawn *S. enterica*—GFP plates at 25°C for 36, 72, 96, or 120 hours. At designated transfer time points, animals were rinsed off *S. enterica*—GFP plates, washed with M9 (4 changes ×15 minutes), concentrated, and then transferred to plates with or without 50 µg/ml Tetracycline that were seeded with *E. coli* HT115 or *S. enterica-*GFP. At designated visualization time points, animals were picked to an NGM—OP50 plate for 1 hour before being picked to a new NGM—OP50 plate. Animals were then visualized at 20× using a Leica MZ FLIII fluorescence stereomicroscope. In heavily colonized animals (heavy) GFP fluorescence was visible in the presence of halogen white light set at 60%, while in weakly infected animals (weak) GFP fluorescence was only visible in the absence of white light. Animals where GFP fluorescence was not detected even in the absence of white light were scored as not infected (none).

### Quantification of intestinal bacterial loads

For the quantification of colony forming units (CFUs), *fer-1(b232ts)* animals were synchronized by treating gravid adults with sodium hydroxide and bleach. About 2,000 synchronized L1 animals were grown on full lawn *S. enterica*—GFP plates at 25°C for 72 hours. At the designated transfer time points, animals were rinsed off *S. enterica*—GFP plates, washed with M9 (4 changes ×15 minutes), concentrated, and then transferred to *S. enterica-*GFP or *E. coli* plus 50 µg/ml Tetracycline plates. At designated CFU time points, animals were picked to 3 NGM—OP50 plates (20 minutes each) before being picked to a 1.5 ml eppendorf tube with 50 µl of PBS plus 0.1% Triton-X-100. A total of 10 animals per condition were mechanically disrupted using a mini-pestle. Serial dilutions of the lysates were spread onto LB/Kanamycin (50 µg/ml) plates to select for *S. enterica*—GFP cells and grown for 24 hours at 37°C.

### Survival assays

Bacteria – *E. coli* HT115 or *S. enterica* were grown overnight for 14 hours in 3 ml LB broth at 37°C. A total of 50 µl (scoring) or 500 µl (exposure) of the resulting cultures were spread onto modified (0.35% peptone) NGM plates with or without 50 µg/ml Tetracycline and allowed to grow for 1–2 days at 25°C to produce a thick lawn. *fer-1(b232ts)* animals were synchronized by treating gravid adults with sodium hydroxide and bleach. Synchronized L1 animals were grown on full lawn *S. enterica*—GFP plates at 25°C for 72 hours before being transferred to the appropriate (treatment or not) plates. The assays were performed at 25°C. Animals were scored every day and were considered dead when they failed to respond to touch. Animals were transferred to fresh plates every other day for the entire length of the experiment. Survival was plotted using Kaplan-Meier survival curves and analyzed by the logrank test using GraphPad Prism (GraphPad Software, Inc., San Diego, CA). Survival curves resulting in *p* values of <0.05 were considered significantly different. A total of 60 animals per condition per experiment were used.

### Longevity assays

*E. coli* was grown as described above. A 50- µl drop of the bacteria was plated on a 3.5-cm plate of modified NGM agar containing 40 µg/ml fluoro-deoxyuridine with or without 50 µg/ml Tetracycline. A total of 100 animals per condition per experiment were used. The assays were performed at 25°C. Survival curves were analyzed as described above.

### RNAi-coupled survival assays

***gsto-1(RNAi)*** **.** *E. coli* HT115(DE3) bacterial strains expressing double-stranded RNA [48] were grown for 9 hours in 5 ml LB broth containing Ampicillin (50 µg/ml) at 37°C. The resulting cultures were seeded onto NGM plates containing Carbenicillin (50 µg/ml) and isopropyl-1-thio-β-D-galactopyranoside (3 mM). dsRNA-expressing bacteria were allowed to grow for 2 days at 25°C to produce a thick lawn. *fer-1(b232ts)* L4 animals were placed on RNAi or vector control plates for 5 days at 15°C until F1 animals developed. *fer-1(b232ts)* F1 L4 animals were placed on a second RNAi or vector control plate and incubated for another 5 days at 15°C until adult F2 animals developed. Gravid F2 RNAi animals were picked to full lawn *E. coli* or *S. enterica*—GFP plates and allowed to lay eggs for 3 hours at 25°C to synchronize a third generation population. These third generation animals were kept on *E. coli* or *S. enterica*—GFP plates for 72 hours before being transferred to plates with or without 50 µg/ml Tetracycline that were seeded with *E. coli* or *S. enterica-*GFP. *unc-22(RNAi)* was used as positive control in all experiments to account for RNAi efficiency. The *gsto-1* (mv_C29E4.7) RNAi vector was verified by DNA sequencing. A total of 60 animals per condition per experiment were scored for survival.

***elt-2(RNAi)*** **and** ***pmk-1(RNAi)*** **.** Production of RNAi plates was the same as described above. Gravid *fer-1(b232ts)* animals were allowed to lay eggs for 3 hours at 25°C on NGM-HT115 plates. Gravid animals were removed and the eggs/plates were incubated for 36 hours at 25°C. Synchronized L4 animals were then transferred to RNAi or vector control plates and incubated for an additional 36 hours at 25°C. Young adult RNAi or vector control animals were then transferred to and grown on full lawn *E. coli* or *S. enterica*—GFP plates for 36 hours at 25°C. Adult worms were then transferred to plates with or without 50 µg/ml Tetracycline that were seeded with *E. coli* or *S. enterica-*GFP. *unc-22(RNAi)* was used as positive control in all experiments to account for RNAi efficiency. The *elt-2* (mv_AAC36130) *and pmk-1* (sjj_B0218.3) RNAi vectors were verified by DNA sequencing. A total of 60 animals per condition per experiment were scored for survival.

### RNA isolation for qRT-PCR

***fer-1(b232ts)*** **.** Animals were synchronized by treating gravid adults with sodium hydroxide and bleach. Synchronized L1 animals were grown on full lawn *E. coli* or *S. enterica*—GFP plates for 72 hours. At 72 hours, animals were rinsed off *E. coli* or *S. enterica*—GFP plates, washed with M9 (4 changes ×15 minutes), concentrated, and then transferred to *E. coli*, *E. coli* plus 50 µg/ml Tetracycline, *E. coli* plus 50 µg/ml Kanamycin, or *S. enterica*—GFP plates. At 24 hours post-transfer, animals were rinsed off plates, washed with M9 (4 changes ×15 minutes), and flash-frozen in Trizol (Life Technologies, Carlsbad, CA). Total RNA was extracted using the RNeasy Plus Universal Kit (Qiagen, Netherlands).

***elt-2(RNAi); fer-1(b232ts)*** **.** Animals were synchronized by treating gravid adults with sodium hydroxide and bleach. Synchronized L1 animals were grown on full lawn *E. coli* plates for 36 hours. At 36 hours, animals were rinsed off *E. coli*, washed with M9 (4 changes ×15 minutes), concentrated, and then placed on RNAi or vector control plates for 36 hours. At 72 hours, animals were rinsed off these plates washed with M9 (4 changes ×15 minutes), concentrated, and then placed on *S. enterica*—GFP plates for 36 hours. At 108 hours, animals were rinsed off *S. enterica*—GFP plates, washed with M9 (4 changes ×15 minutes), concentrated, and then transferred to *E. coli*, *E. coli* plus 50 µg/ml Tetracycline, or *S. enterica*—GFP plates for 24 hours. Animals were rinsed off plates, washed with M9 (4 changes ×15 minutes), and flash-frozen in Trizol (Life Technologies, Carlsbad, CA). Total RNA was extracted using the RNeasy Plus Universal Kit (Qiagen, Netherlands). All studies were performed at 25°C.

### Quantitative Real-Time PCR (qRT-PCR)

Total RNA was obtained as described above. A total of 1 µg total RNA was oligo(dT) primed and reverse transcribed in a 50 µl volume using the SuperScript III First-Strand Synthesis System (Life Technologies, Carlsbad, CA). Reactions without the addition of reverse transcriptase (RT) were also performed and served as controls for contaminating genomic DNA in quantitative PCR experiments. Two µl of the resulting plus or minus RT reactions served as templates in quantitative PCR experiments using Power SYBR Green PCR Master Mix (Life Technologies, Carlsbad, CA) and the StepOnePlus Real-Time PCR System (Life Technologies, Carlsbad, CA). For each sample, 3 technical replicates were performed. Pan-actin-normalized Ct values were determined using the StepOnePlus Software (Life Technologies, Carlsbad, CA). Primer sequences are available upon request. When applicable a one or two variable *t*-test was performed.

### RNA isolation for microarray analysis

*fer-1(b232ts)* animals were synchronized by treating gravid adults with sodium hydroxide and bleach. Synchronized L1 animals were grown on full lawn *E. coli* OP50 (uninfected) or full lawn *S. enterica* plates at 25°C for 36, 72, 96, or 120 hours. At designated transfer time points, animals were rinsed off *S. enterica* plates, washed with M9 (4 changes ×15 minutes), concentrated, and then transferred to *S. enterica* or *E. coli* plus 50 µg/ml Tetracycline plates. At designated harvesting time points, animals were rinsed off plates, washed with M9 (4 changes ×15 minutes), and flash-frozen in Trizol (Life Technologies, Carlsbad, CA). Total RNA was extracted using the RNeasy Plus Universal Kit (Qiagen, Netherlands).

### Microarray analysis

Total RNA was assessed for quality with an Agilent 2100 Bioanalyzer G2939A (Agilent Technologies, Santa Clara, CA) and a Nanodrop 8000 spectrophotomer (Thermo, Wilmington, DE). 100 ng of total RNA was converted to 1.65 µg Cy-3-labeled, linearly amplified cRNA using the Low Input Quick Amp (LIQA) Labeling One-Color Microarray-Based Gene Expression Analysis Kit (Agilent Technologies, Santa Clara, CA). cRNA was fragmented and added to 44 K feature Agilent *C. elegans* Gene Expression Microarray V2 slides (Agilent Technologies, Santa Clara, CA). Hybridization was performed in the Agilent rotisserie Hybridization Oven for 17 hours. Arrays were subsequently washed and scanned with the Agilent B scanner according to standard Agilent protocols (Agilent Technologies, Santa Clara, CA). Scanned data was log2 transformed and quantile normalized using Partek Genomics Suite (St. Louis, MO). Analysis of variance (ANOVA) t tests and fold-change calculations were also performed using Partek Genomics Suite (St. Louis, MO). For each of the 5 time points, 2 biological replicates were assessed. The microarray data was deposited in the Gene Expression Omnibus database: GSE54212.

### Bioinformatics

Gene lists were culled from the literature and passed through WormBase Converter [52] using the WS220 genome release as the output (references are noted in Table S5). A total of 20,834 WS220 genes are represented by 1 or more probes in the Agilent *C. elegans* V2 array (Agilent Technologies, Santa Clara, CA). Gene ontology analysis was performed using the DAVID Bioinformatics Database (david.abcc.ncifcrf.gov/). The most significant gene ontology term in each DAVID functional annotation cluster was set as the significance of the overall cluster. Statistical significance of the overlap between two gene sets was calculated using the following on-line program: nemates.org/MA/progs/overlap_stats.html. Representation Factor represents the number of overlapping genes divided by the expected number of overlapping genes drawn from 2 independent groups. A background gene list of 20,834 was used for the calculation. P values were calculated using the hypergeometric probability. 1.5 kb cis-regulatory sequences were identified using WormMart (wormbase.org). Expression patterns were determined using WormMine (wormbase.org). Detailed bioinformatics protocols are available upon request.

## Supporting Information

Figure S1 Tetracycline effectively limits progression of an *S. enterica* infection. *fer-1(b232ts)* L1 animals were exposed to *S. enterica*—GFP for 72 hours and transferred to the indicated bacteria-antibiotic plates for 48 hours. Overall GFP intensity in the intestinal lumen was determined using an MZFLIII Leica stereomicroscope. Three levels of colonization were determined as heavy, weak, or none as described in Materials and Methods. The mean of 2 plates is shown. For each condition, we assayed 20–40 animals.
(TIF)

Figure S2 Survival of uninfected animals exposed to Tetracycline. *fer-1(b232ts)* L1 animals were grow on killed *E. coli* for 72 hours and then transferred to killed *E. coli* or killed *E. coli* plus Tetracycline and scored for survival. Animals were scored for survival 72 hours after the initial exposure to *E. coli*. Plates containing 40 µg/ml 5-fluorodeoxyuridine were used, which is a standard method in nematode aging research. N = 100 animals per condition. The graphs represent the combined results of 2 independent experiments.
(TIF)

Figure S3 Gene expression changes in infected animals treated with Kanamycin mimic gene expression changes in infected animals treated with Tetracycline. (A–B) Transcript levels of 4 selected down-regulated genes (B) and 5 selected up-regulated genes (C) as determined using qRT-PCR. Black striped bars represent gene expression changes in L1 animals grown on *E. coli* for 72 hours and then treated with Kanamycin for 24 hours relative to L1 animals grown on *E. coli* for 96 hours. Gray striped bars represent gene expression changes in L1 animals grown on *S. enterica* for 72 hours and then treated with Kanamycin for 24 hours relative to animals grown on *S. enterica* for 96 hours. (C–D) Comparison of gene expression changes in 4 selected down-regulated genes (C) and 5 selected up-regulated genes (D) during recovery with Tetracycline or Kanamycin. Gray bars represent gene expression changes in L1 animals grown on *S. enterica* for 72 hours and then treated with Tetracycline for 24 hours relative to animals grown on *S. enterica* for 96 hours. Gray striped bars represent gene expression changes in L1 animals grown on *S. enterica* for 72 hours and then treated with Kanamycin for 24 hours relative to animals grown on *S. enterica* for 96 hours. qRT-PCR studies were performed in duplicate. SEM is shown.
(TIF)

Figure S4 *gsto-1(RNAi)* animals are minimally affected during resolution of an infection. (A) Control *fer-1(b232ts)* or *fer-1(b232ts) gsto-1(RNAi)* L1 animals were exposed to *E. coli* or *S. enterica*—GFP for 72 hours and then transferred to *E. coli* plus Tetracycline or *S. enterica*—GFP and scored for survival. (B) Control *fer-1(b232ts)* or *fer-1(b232ts) gsto-1(RNAi)* animals were exposed to *E. coli* or *S. enterica*—GFP for 72 hours and then transferred to *E. coli* plus Tetracycline and scored for survival. N = 60 animals per condition. The graphs represent the combined results of 5 independent experiments.
(TIF)

Figure S5 ELT-2-controlled immunity and structural genes that are not significantly altered during recovery from *S. enterica* infection. (A–B) Transcript levels of ELT-2-regulated immunity genes (A) or ELT-2-regulated intestinal homeostasis genes (B) over the infection time course. The expression values of animals grown on *S. enterica* for 72 hours and then treated with Tetracycline for 24 hours are denoted with open circles. The expression values of animals grown on *S. enterica* for 96 hours are denoted with an X. (C) Transcript levels of *clec-67* as determined using qRT-PCR. Black bars represent gene expression changes in L1 animals grown on *E. coli* for 72 hours and then treated with Tetracycline for 24 hours relative to L1 animals grown on *E. coli* for 96 hours. Gray bars represent gene expression changes in L1 animals grown on *S. enterica* for 72 hours and then treated with Tetracycline for 24 hours relative to animals grown on *S. enterica* for 96 hours. qRT-PCR studies were performed in triplicate. SEM is shown.
(TIF)

Figure S6 Survival of *elt-2(RNAi)* animals exposed to Tetracycline is not affected. Control *fer-1(b232ts)* or *fer-1(b232ts) elt-2(RNAi)* animals were exposed to *E. coli* or *S. enterica*—GFP for 36 hours and then transferred to *E. coli* or *S. enterica*—GFP and scored for survival. N = 20–60 animals per condition. The graphs represent the combined results of 2 independent experiments.
(TIF)

Table S1 Microarray expression data.
(XLSX)

Table S2 Genes altered more than 2-fold (p<0.05) upon recovery using microarray profiling.
(XLSX)

Table S3 GO enrichment analysis using DAVID bioinformatics database.
(XLSX)

Table S4 Genes altered more than 2-fold (p<0.05) upon recovery at both the 96-hour timepoint and the 120-hour timepoint.
(XLSX)

Table S5 Overlap of recovery gene sets and previously studied gene sets.
(XLSX)

Table S6 Analysis of TGATAA sites in 1.5kb promoter regions of qRT-PCR-confirmed recovery genes.
(XLSX)

Table S7 Expression patterns of recovery genes via WormMine.
(XLSX)
