# Correction: Recovery from an Acute Infection in *C*. *elegans* Requires the GATA Transcription Factor ELT-2

**DOI:** 10.1371/journal.pgen.1005100

**Published:** 2015-04-10

**Authors:** The PLOS Genetics Staff

“The legend for Figure 5 is incorrect. The correct legend is:

**Fig 5:**
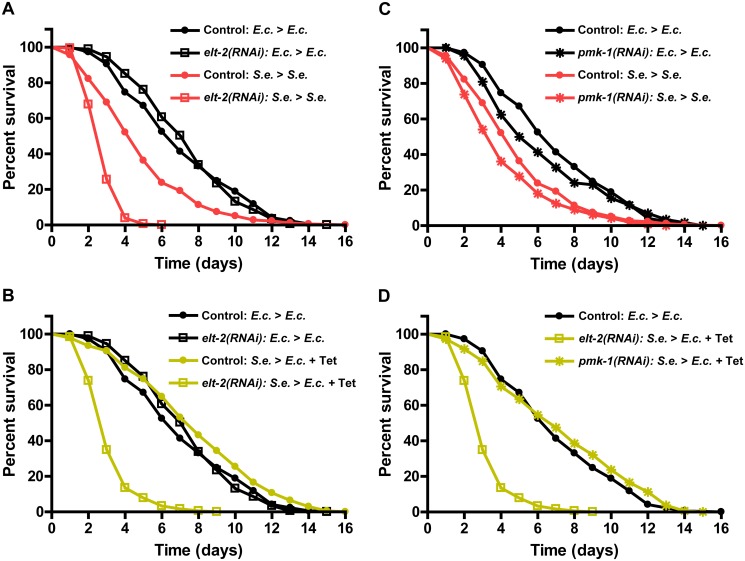
elt-2(RNAi) animals are unable to resolve an infection. (A) Control fer-1(b232ts) or fer-1(b232ts) elt-2(RNAi) young adult animals were exposed to E. coli or S. enterica—GFP for 36 hours and then transferred to E. coli or S. enterica—GFP and scored for survival. (B) Control fer-1(b232ts) or fer-1(b232ts) elt-2(RNAi) animals were exposed to E. coli or S. enterica—GFP for 36 hours and then transferred to E. coli or E. coli plus Tetracycline and scored for survival. (C) Control fer-1(b232ts) or fer-1(b232ts) pmk-1(RNAi) young adult animals were exposed to E. coli or S. enterica—GFP for 36 hours and then transferred to E. coli or S. enterica—GFP and scored for survival. (D) Control fer-1(b232ts), fer-1(b232ts) elt-2(RNAi), or fer-1(b232ts) pmk-1(RNAi) young adult animals were exposed to E. coli or S. enterica—GFP for 36 hours and then transferred to E. coli or E. coli plus Tetracycline and scored for survival. N = 60 animals per condition. The graphs represent the combined results of 3 independent experiments.

## Supporting Information

Figure S1 is labeled incorrectly. The second column should be labeled “E. coli OP50” and the third column should be labeled “E.c. OP50 Amp(10)”. The correct legend is:

S1 Fig Tetracycline effectively limits progression of an S. enterica infection. fer-1(b232ts) L1 animals were exposed to S. enterica—GFP for 72 hours and transferred to the indicated bacteria-antibiotic plates for 48 hours. Overall GFP intensity in the intestinal lumen was determined using an MZFLIII Leica stereomicroscope. Three levels of colonization were determined as heavy, weak, or none as described in Materials and Methods. The mean of 2 plates is shown. For each condition, we assayed 20–40 animals.
(TIF)

The legend for Figure S3 is incorrect. The correct legend is:

S3 Fig Gene expression changes in infected animals treated with Kanamycin mimic gene expression changes in infected animals treated with Tetracycline. (A-B) Transcript levels of 4 selected down-regulated genes (A) and 5 selected up-regulated genes (B) as determined using qRT-PCR. Black striped bars represent gene expression changes in L1 animals grown on E. coli for 72 hours and then treated with Kanamycin for 24 hours relative to L1 animals grown on E. coli for 96 hours. Gray striped bars represent gene expression changes in L1 animals grown on S. enterica for 72 hours and then treated with Kanamycin for 24 hours relative to animals grown on S. enterica for 96 hours. (C-D) Comparison of gene expression changes in 4 selected down-regulated genes (C) and 5 selected up-regulated genes (D) during recovery with Tetracycline or Kanamycin. Gray bars represent gene expression changes in L1 animals grown on S. enterica for 72 hours and then treated with Tetracycline for 24 hours relative to animals grown on S. enterica for 96 hours. Gray striped bars represent gene expression changes in L1 animals grown on S. enterica for 72 hours and then treated with Kanamycin for 24 hours relative to animals grown on S. enterica for 96 hours. qRT-PCR studies were performed in duplicate. SEM is shown.
(TIF)
