# Supplementary figures and images for: Recovery from an Acute Infection in C. elegans Requires the GATA Transcription Factor ELT-2
Source: PLoS Genet. 2014 Oct 23;10(10):e1004609. doi: 10.1371/journal.pgen.1004609 (PMC4207467; doi:10.1371/journal.pgen.1004609)

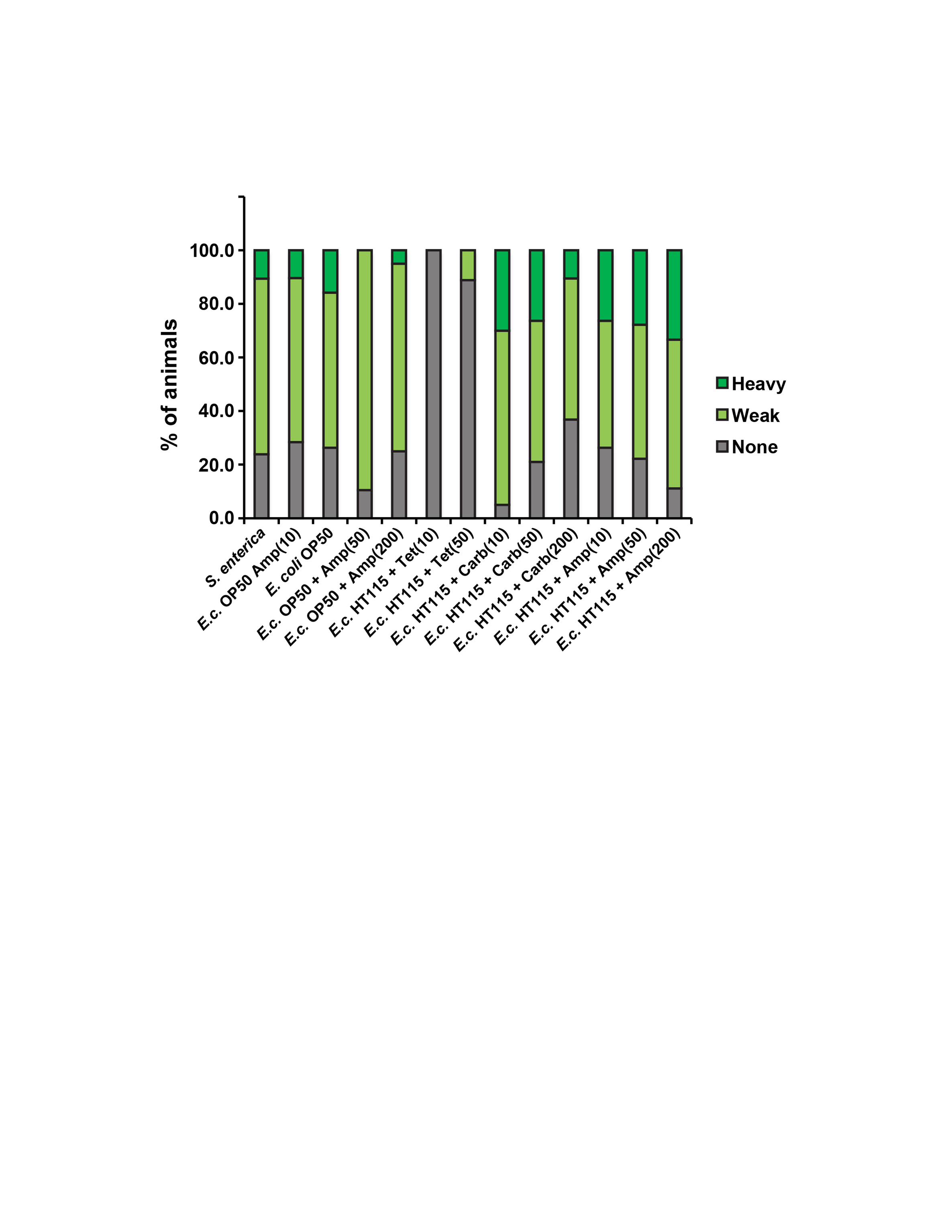

Supplement: Figure S1 — Tetracycline effectively limits progression of an S. enterica infection. fer-1(b232ts) L1 animals were exposed to S. enterica—GFP for 72 hours and transferred to the indicated bacteria-antibiotic plates for 48 hours. Overall GFP intensity in the intestinal lumen was determined using an MZFLIII Leica stereomicroscope. Three levels of colonization were determined as heavy, weak, or none as described in Materials and Methods. The mean of 2 plates is shown. For each condition, we assayed 20–40 animals. (TIF) [file pgen.1004609.s001.tif]

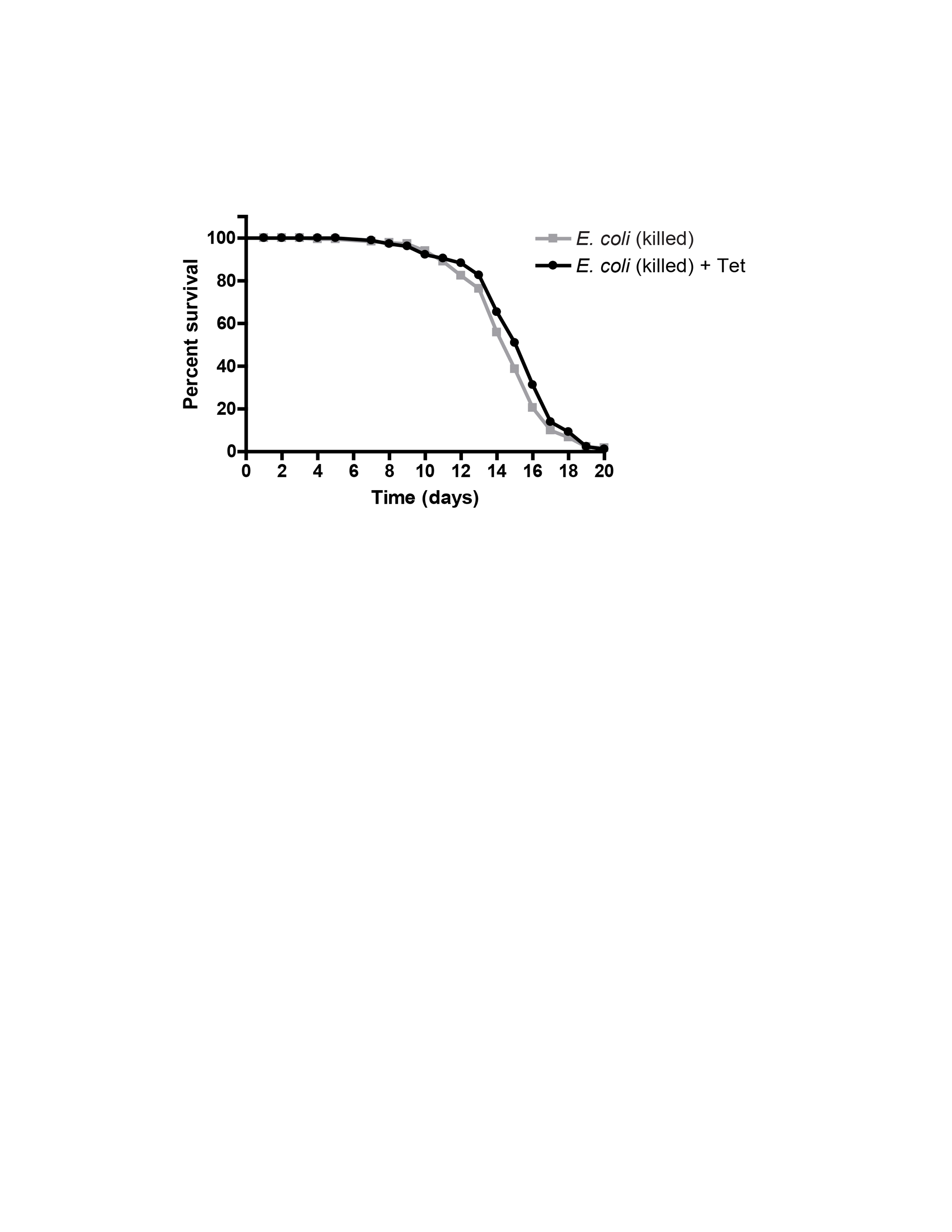

Supplement: Figure S2 — Survival of uninfected animals exposed to Tetracycline. fer-1(b232ts) L1 animals were grow on killed E. coli for 72 hours and then transferred to killed E. coli or killed E. coli plus Tetracycline and scored for survival. Animals were scored for survival 72 hours after the initial exposure to E. coli. Plates containing 40 µg/ml 5-fluorodeoxyuridine were used, which is a standard method in nematode aging research. N = 100 animals per condition. The graphs represent the combined results of 2 independent experiments. (TIF) [file pgen.1004609.s002.tif]

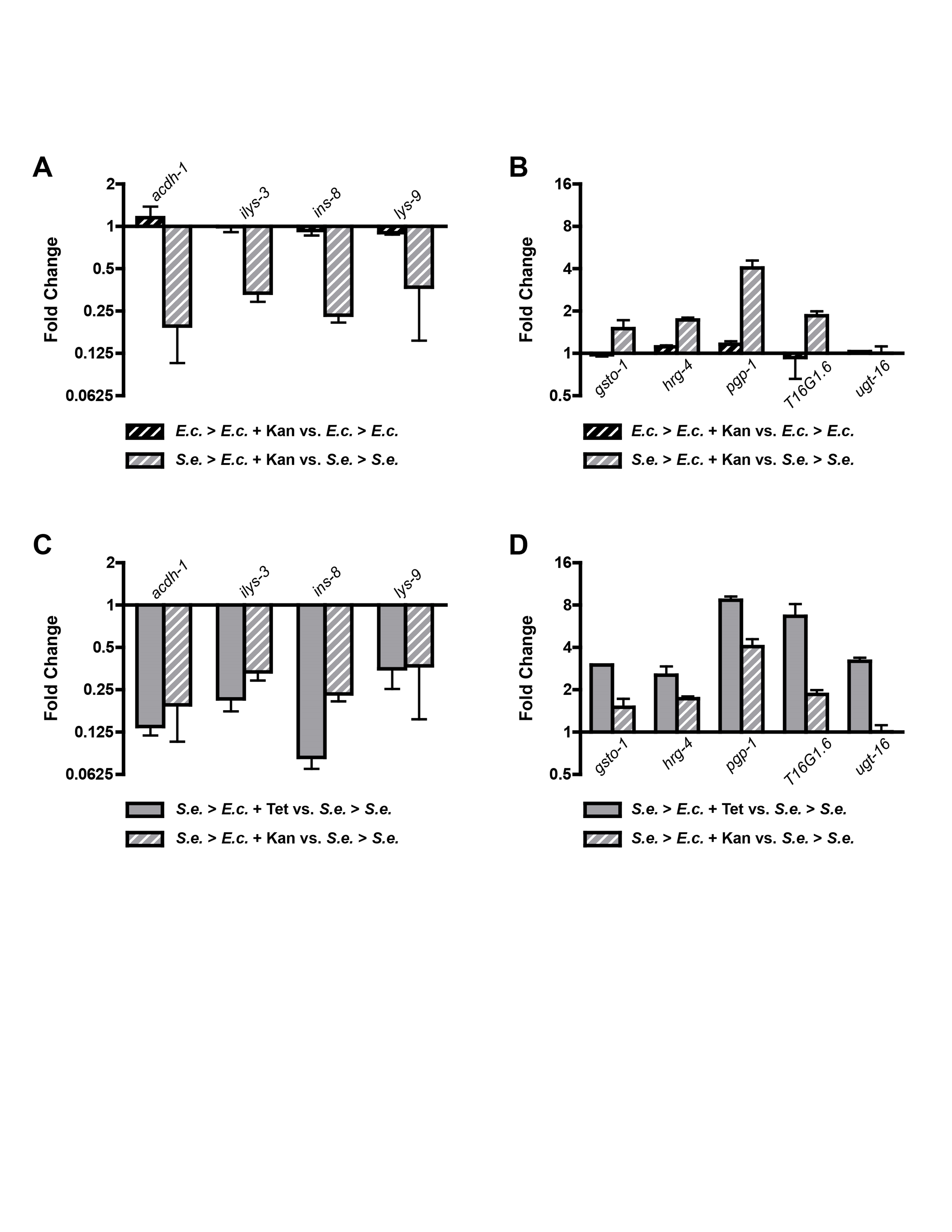

Supplement: Figure S3 — Gene expression changes in infected animals treated with Kanamycin mimic gene expression changes in infected animals treated with Tetracycline. (A–B) Transcript levels of 4 selected down-regulated genes (B) and 5 selected up-regulated genes (C) as determined using qRT-PCR. Black striped bars represent gene expression changes in L1 animals grown on E. coli for 72 hours and then treated with Kanamycin for 24 hours relative to L1 animals grown on E. coli for 96 hours. Gray striped bars represent gene expression changes in L1 animals grown on S. enterica for 72 hours and then treated with Kanamycin for 24 hours relative to animals grown on S. enterica for 96 hours. (C–D) Comparison of gene expression changes in 4 selected down-regulated genes (C) and 5 selected up-regulated genes (D) during recovery with Tetracycline or Kanamycin. Gray bars represent gene expression changes in L1 animals grown on S. enterica for 72 hours and then treated with Tetracycline for 24 hours relative to animals grown on S. enterica for 96 hours. Gray striped bars represent gene expression changes in L1 animals grown on S. enterica for 72 hours and then treated with Kanamycin for 24 hours relative to animals grown on S. enterica for 96 hours. qRT-PCR studies were performed in duplicate. SEM is shown. (TIF) [file pgen.1004609.s003.tif]

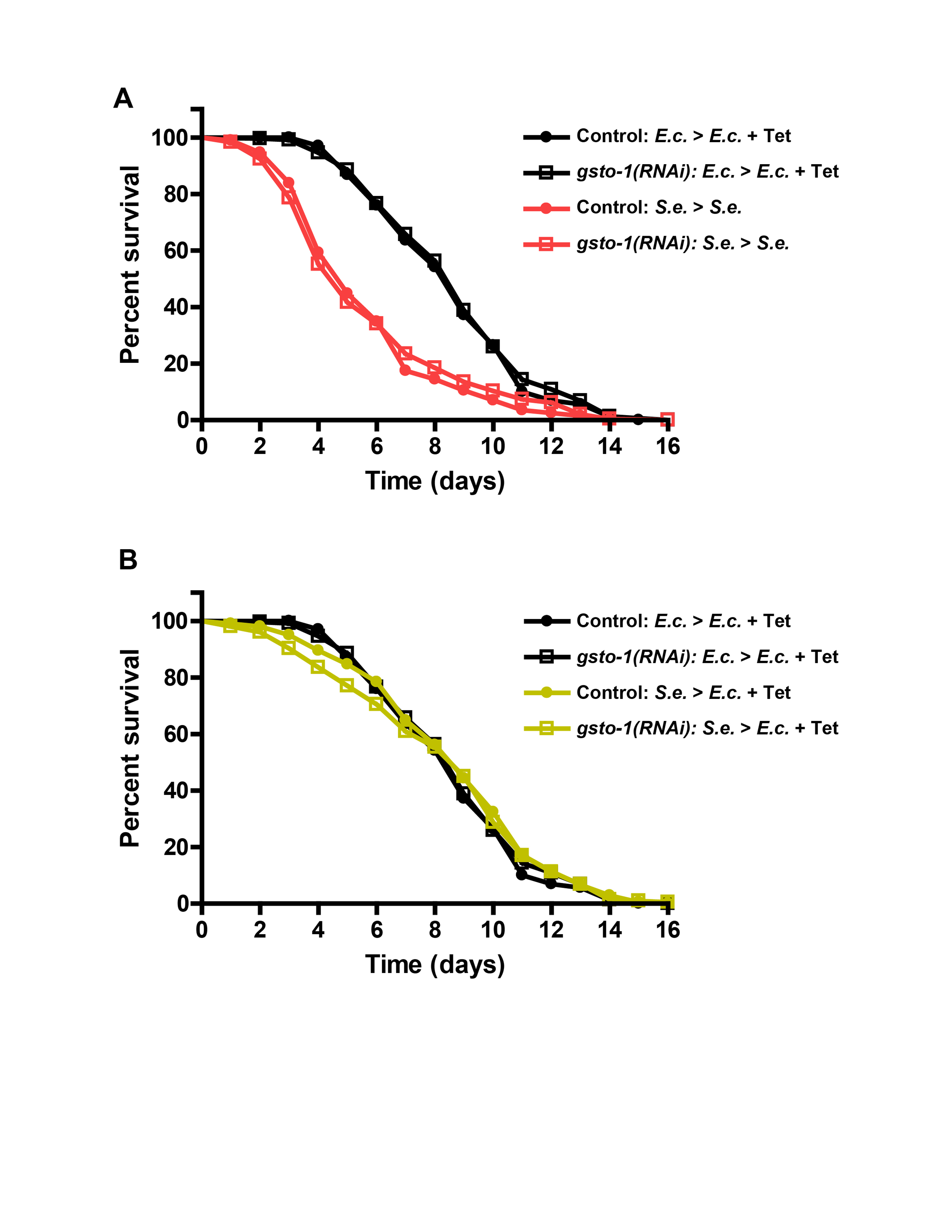

Supplement: Figure S4 — gsto-1(RNAi) animals are minimally affected during resolution of an infection. (A) Control fer-1(b232ts) or fer-1(b232ts) gsto-1(RNAi) L1 animals were exposed to E. coli or S. enterica—GFP for 72 hours and then transferred to E. coli plus Tetracycline or S. enterica—GFP and scored for survival. (B) Control fer-1(b232ts) or fer-1(b232ts) gsto-1(RNAi) animals were exposed to E. coli or S. enterica—GFP for 72 hours and then transferred to E. coli plus Tetracycline and scored for survival. N = 60 animals per condition. The graphs represent the combined results of 5 independent experiments. (TIF) [file pgen.1004609.s004.tif]

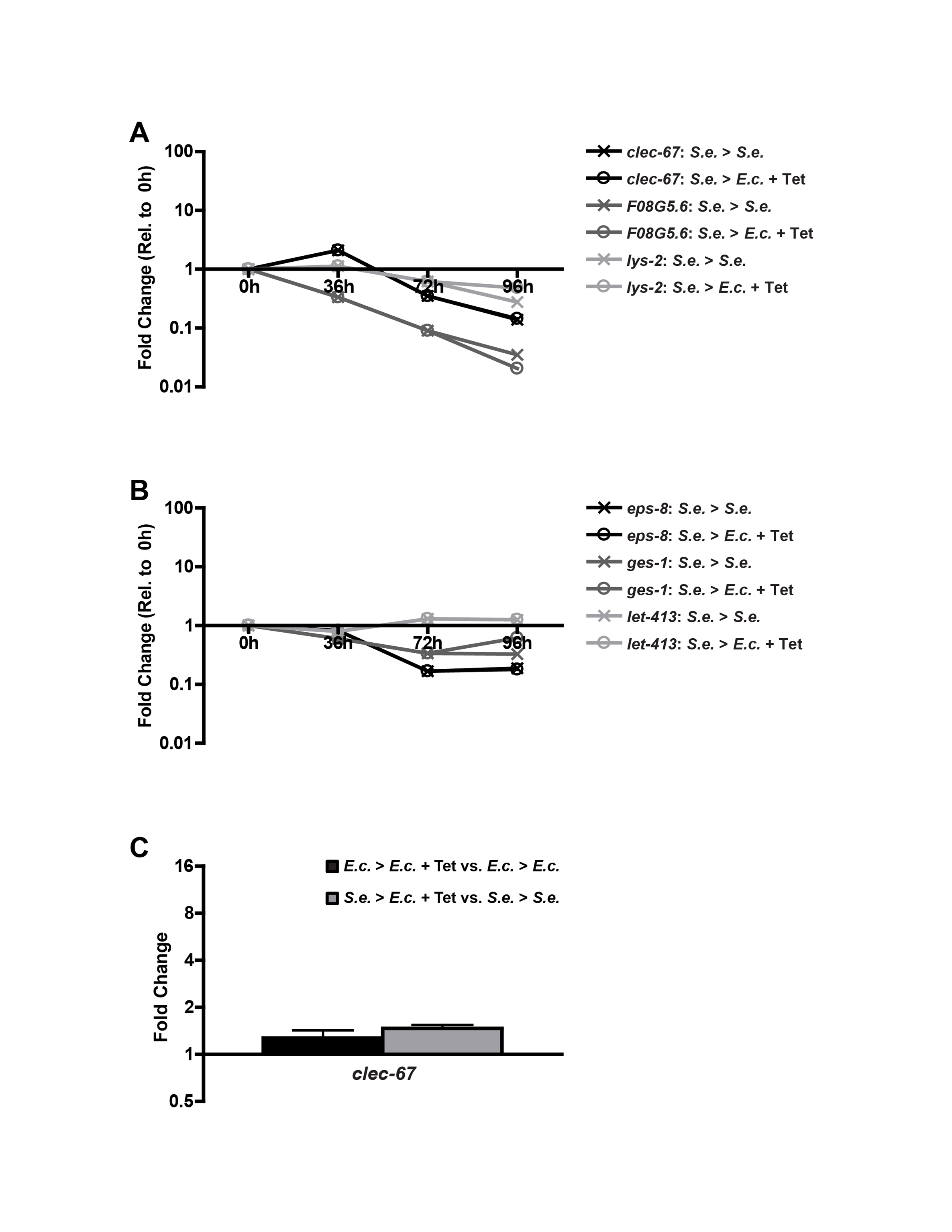

Supplement: Figure S5 — ELT-2-controlled immunity and structural genes that are not significantly altered during recovery from S. enterica infection. (A–B) Transcript levels of ELT-2-regulated immunity genes (A) or ELT-2-regulated intestinal homeostasis genes (B) over the infection time course. The expression values of animals grown on S. enterica for 72 hours and then treated with Tetracycline for 24 hours are denoted with open circles. The expression values of animals grown on S. enterica for 96 hours are denoted with an X. (C) Transcript levels of clec-67 as determined using qRT-PCR. Black bars represent gene expression changes in L1 animals grown on E. coli for 72 hours and then treated with Tetracycline for 24 hours relative to L1 animals grown on E. coli for 96 hours. Gray bars represent gene expression changes in L1 animals grown on S. enterica for 72 hours and then treated with Tetracycline for 24 hours relative to animals grown on S. enterica for 96 hours. qRT-PCR studies were performed in triplicate. SEM is shown. (TIF) [file pgen.1004609.s005.tif]

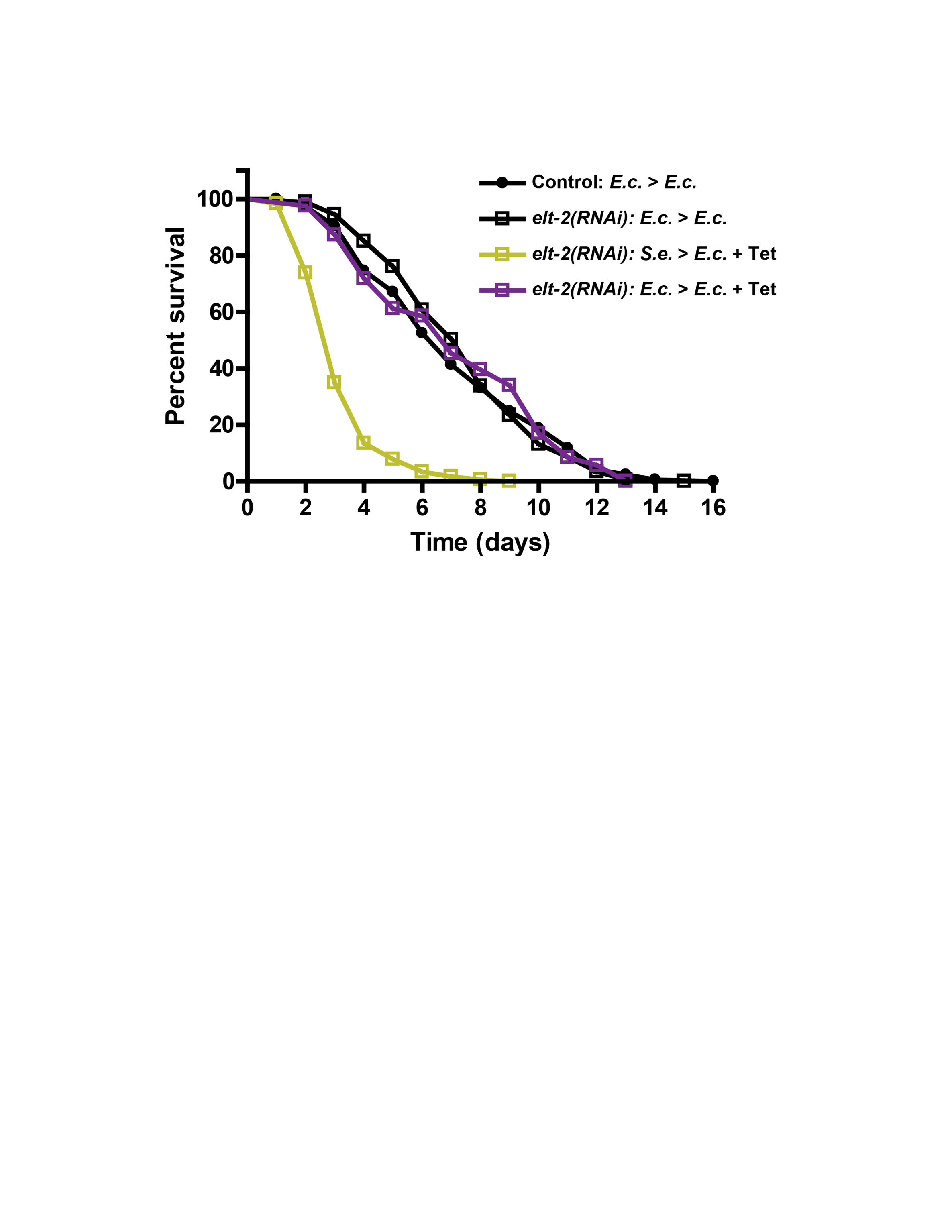

Supplement: Figure S6 — Survival of elt-2(RNAi) animals exposed to Tetracycline is not affected. Control fer-1(b232ts) or fer-1(b232ts) elt-2(RNAi) animals were exposed to E. coli or S. enterica—GFP for 36 hours and then transferred to E. coli or S. enterica—GFP and scored for survival. N = 20–60 animals per condition. The graphs represent the combined results of 2 independent experiments. (TIF) [file pgen.1004609.s006.tif]
