# Supplementary figures and images for: Correction: Recovery from an Acute Infection in C. elegans Requires the GATA Transcription Factor ELT-2
Source: PLoS Genet. 2015 Apr 10;11(4):e1005100. doi: 10.1371/journal.pgen.1005100 (PMC4393128; doi:10.1371/journal.pgen.1005100)

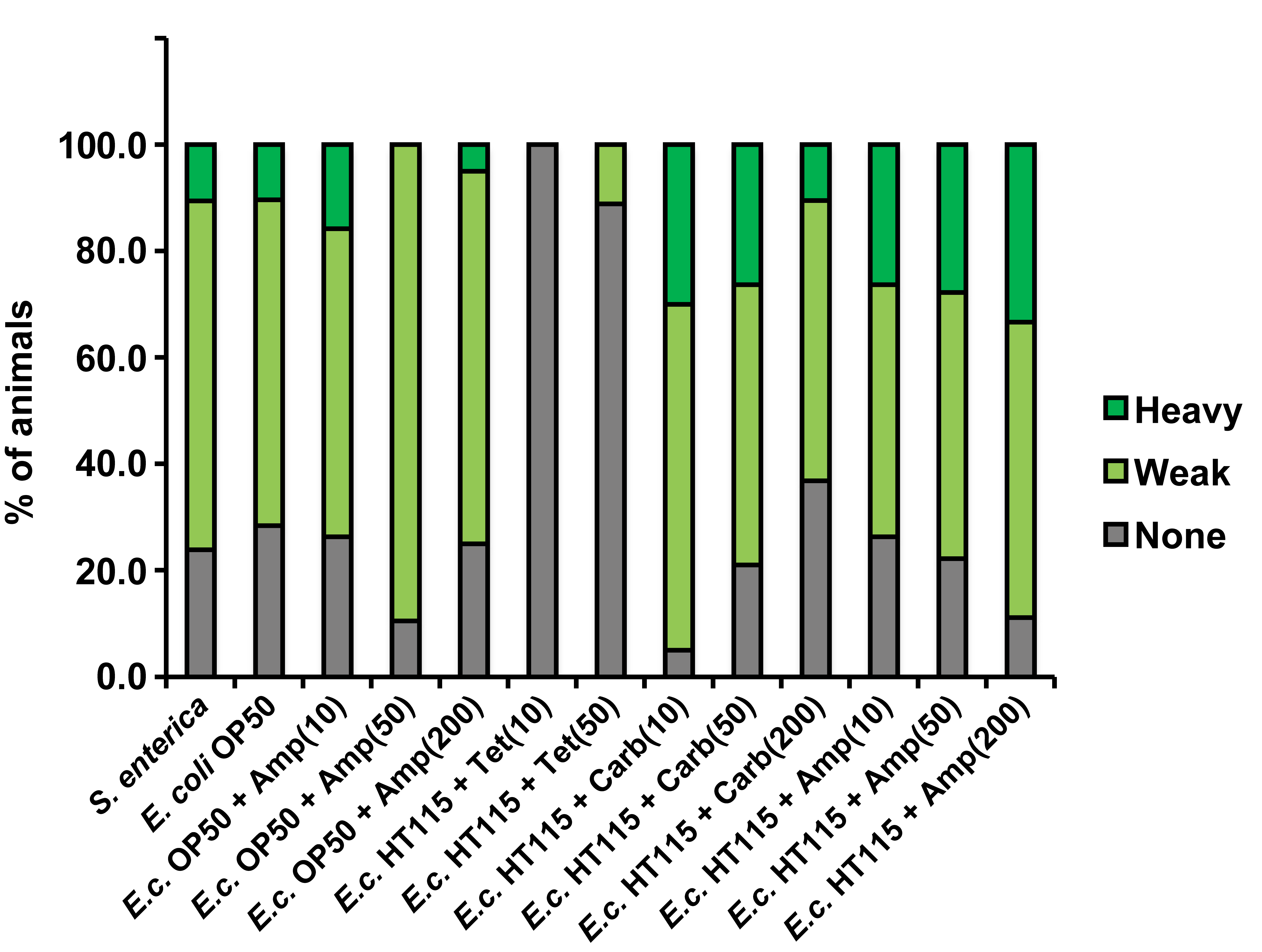

Supplement: S1 Fig — fer-1(b232ts) L1 animals were exposed to S. enterica—GFP for 72 hours and transferred to the indicated bacteria-antibiotic plates for 48 hours. Overall GFP intensity in the intestinal lumen was determined using an MZFLIII Leica stereomicroscope. Three levels of colonization were determined as heavy, weak, or none as described in Materials and Methods. The mean of 2 plates is shown. For each condition, we assayed 20–40 animals. (TIF) [file pgen.1005100.s001.tif]

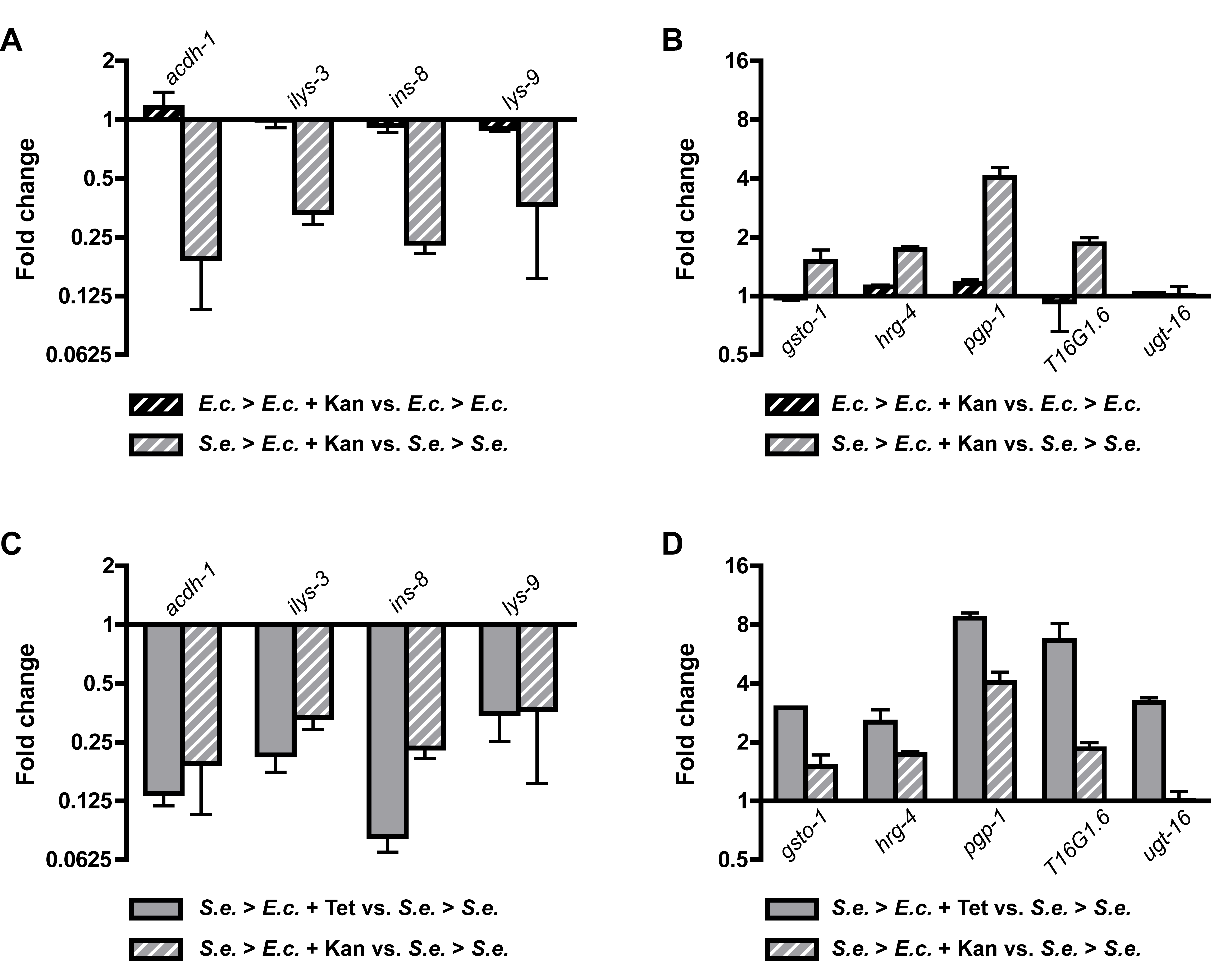

Supplement: S3 Fig — (A-B) Transcript levels of 4 selected down-regulated genes (A) and 5 selected up-regulated genes (B) as determined using qRT-PCR. Black striped bars represent gene expression changes in L1 animals grown on E. coli for 72 hours and then treated with Kanamycin for 24 hours relative to L1 animals grown on E. coli for 96 hours. Gray striped bars represent gene expression changes in L1 animals grown on S. enterica for 72 hours and then treated with Kanamycin for 24 hours relative to animals grown on S. enterica for 96 hours. (C-D) Comparison of gene expression changes in 4 selected down-regulated genes (C) and 5 selected up-regulated genes (D) during recovery with Tetracycline or Kanamycin. Gray bars represent gene expression changes in L1 animals grown on S. enterica for 72 hours and then treated with Tetracycline for 24 hours relative to animals grown on S. enterica for 96 hours. Gray striped bars represent gene expression changes in L1 animals grown on S. enterica for 72 hours and then treated with Kanamycin for 24 hours relative to animals grown on S. enterica for 96 hours. qRT-PCR studies were performed in duplicate. SEM is shown. (TIF) [file pgen.1005100.s002.tif]
